# German Mobile Apps in Rheumatology: Review and Analysis Using the Mobile Application Rating Scale (MARS)

**DOI:** 10.2196/14991

**Published:** 2019-08-05

**Authors:** Johannes Knitza, Koray Tascilar, Eva-Maria Messner, Marco Meyer, Diana Vossen, Almut Pulla, Philipp Bosch, Julia Kittler, Arnd Kleyer, Philipp Sewerin, Johanna Mucke, Isabell Haase, David Simon, Martin Krusche

**Affiliations:** 1 Department of Internal Medicine 3 – Rheumatology and Immunology Friedrich-Alexander University Erlangen-Nürnberg University Hospital Erlangen Erlangen Germany; 2 Working Group Young Rheumatology German Society for Rheumatology Berlin Germany; 3 Department for Clinical Psychology and Psychotherapy Ulm University Ulm Germany; 4 Rheumatologie, Klinische Immunologie, Nephrologie Asklepios Klinik Altona Hamburg Germany; 5 Rheinisches Rheumazentrum Meerbusch St Elisabeth Hospital Meerbusch Germany; 6 Department of Rheumatology and Immunology Medical University Graz Graz Austria; 7 Department of Rheumatology and Hiller Research Unit Rheumatology Heinrich Heine University Düsseldorf Germany; 8 Department of Rheumatology and Clinical Immunology Charité Universitätsmedizin Berlin Germany

**Keywords:** mobile apps, eHealth, rheumatology, mHealth, Mobile Application Rating Scale

## Abstract

**Background:**

Chronic rheumatic diseases need long-term treatment and professional supervision. Mobile apps promise to improve the lives of patients and physicians. In routine practice, however, rheumatology apps are largely unknown and little is known about their quality and safety.

**Objective:**

The aim of this study was to provide an overview of mobile rheumatology apps currently available in German app stores, evaluate app quality using the Mobile Application Rating Scale (MARS), and compile brief, ready-to-use descriptions for patients and rheumatologists.

**Methods:**

The German App Store and Google Play store were systematically searched to identify German rheumatology mobile apps for patient and physician use. MARS was used to independently assess app quality by 8 physicians, 4 using Android and 4 using iOS smartphones. Apps were randomly assigned so that 4 apps were rated by all raters and the remaining apps were rated by two Android and two iOS users. Furthermore, brief app descriptions including app developers, app categories, and features were compiled to inform potential users and developers.

**Results:**

In total, 128 and 63 apps were identified in the German Google Play and App Store, respectively. After removing duplicates and only including apps that were available in both stores, 28 apps remained. Sixteen apps met the inclusion criteria, which were (1) German language, (2) availability in both app stores, (3) targeting patients or physicians as users, and (4) clearly including rheumatology or rheumatic diseases as subject matter. Exclusion criteria were (1) congress apps and (2) company apps with advertisements. Nine apps addressed patients and 7 apps addressed physicians. No clinical studies to support the effectiveness and safety of apps could be found. Pharmaceutical companies were the main developers of two apps. Rheuma Auszeit was the only app mainly developed by a patient organization. This app had the highest overall MARS score (4.19/5). Three out of 9 patient apps featured validated questionnaires. The median overall MARS score was 3.85/5, ranging from 2.81/5 to 4.19/5. One patient-targeted and one physician-targeted app had MARS scores >4/5. No significant rater gender or platform (iOS/Android) differences could be observed. The overall correlation between app store ratings and MARS scores was low and inconsistent between platforms.

**Conclusions:**

To our knowledge, this is the first study that systematically identified and evaluated mobile apps in rheumatology for patients and physicians available in German app stores. We found a lack of supporting clinical studies, use of validated questionnaires, and involvement of academic developers. Overall app quality was heterogeneous. To create high-quality apps, closer cooperation led by patients and physicians is vital.

## Introduction

There is great potential in using eHealth tools, especially in chronic rheumatic diseases [1]. In the anticipated reality of Rheumatology 4.0 computer-aided diagnostic systems allowing precise and quick diagnosis [2], mobile apps and other eHealth tools could improve the positions of all stakeholders, including patients, physicians, health insurance companies, and the pharmaceutical industry.

The use of diagnostic decision support systems could shorten the time to correct diagnosis, even for rare diseases [3]. Once a correct diagnosis is established, patients and physicians need to maintain disease control, which necessitates continuous monitoring of treatment adherence, accurate symptom tracking, and surveillance of adverse treatment effects. eHealth is promising to increase the quantity, quality, and availability of medical data, thus allowing a more precise and personalized treatment. A recent study showed that remote monitoring of disease activity using physical activity trackers precisely detects flareups in patients with rheumatoid arthritis [4]. This is a good example in which an accurate clinical assessment is accomplished using an eHealth tool without necessitating a direct patient-physician encounter. Such tools could drastically increase the efficiency of health care delivery.

The development of apps is becoming easier and less expensive thanks to the lack of restrictions on interventions in app stores. These low market barriers attract various businesses that seek to seize the opportunity of entering the profitable health care market [5]. This leads to considerable heterogeneity regarding security and quality in general.

Quality indicators for health care–related apps beyond the app store star ratings, comments, and number of downloads are largely unavailable. Trust marks and certification labels (like CE marking indicating conformity with health, safety, and environmental protection standards for products sold within the European Economic Area) for apps are rarely found [6], making quality assessment of an app a challenge for the end user. A number of tools have been proposed to this end [7,8]. Among the relatively established tools to rate app quality is the validated Mobile Application Rating Scale (MARS) [9]. Since its publication in 2015, it has been used to rate various medical mobile apps [10,11]. The MARS score is based on a 5-point Likert scale in four sections with multiple items: engagement (5 items), functionality (4 items), aesthetics (3 items), and information quality (7 items). In addition, there is a subjective section consisting of 4 items.

A New Zealand study recently reported the results of a MARS assessment evaluating patient apps for rheumatoid arthritis and found a lack of high-quality apps [10]. Such systematic quality assessments are scarce and represent an unmet need. A recent survey conducted by the Working Group Young Rheumatology of the German Society for Rheumatology (Arbeitsgemeinschaft Junge Rheumatologie, or rheumadocs) showed that medical app use among German rheumatologists increased by 12% during two years, yet rheumatologists were aware of only two recommendable apps specific to rheumatology (RheumaHelper, RheumaLive) [5].

To our knowledge, no systematic quality assessment of rheumatology apps available in German app stores has yet been carried out. Therefore, the aim of this study was to identify and evaluate rheumatology-specific German mobile apps targeting patients or physicians.

## Methods

### Selection of Mobile Apps

An extensive German App Store and Google Play search was performed from May 1-31, 2018. This search included the following search terms: “Rheuma” OR “Rheumatologie” OR “Arthritis” OR “Psoriasisarthritis” OR “PsA” OR “PsoA” OR “Rheumatoide Arthritis” OR “RA” OR “Morbus Bechterew” OR “Spondylarthritis” OR “Spondylitis ankylosans” OR “Spondylopathie” OR “axSpa” OR “Spondylarthropathie” OR “Spa” OR “Spondylitis ankylosans” OR “ankylosierende Spondylarthritis” OR “Kollagenose” OR “SLE” OR “Systemischer Lupus Erythematodes” OR “Sklerodermie” OR “Lupus” OR “Sjögren” OR “APS” OR “Antiphospholipidsyndrom” OR “systemische Sklerose” OR “SSc” OR “Polymyositis” OR “Dermatomyositis” OR “RZA” OR “Riesenzellarteriitis” OR “Riesenzellarteritis” OR “EGPA” OR “GPA” OR “eosinophile Granulomatose mit Polyangiitis” OR “Granulomatose mit Polyangiitis” OR “PAN” OR “panarteriitis nodosa” OR “polyarteriitis nodosa” OR “mikroskopische Polyangiitis” OR “Morbus Behcet” OR ”Takayasu Arteriitis” OR “Kawasaki Syndrom” OR “Arteriitis temporalis” OR “PMR” OR “Polymyalgie” OR “Polymyalgia rheumatica” OR “reaktive Arthritis” OR “enteropathische Arthritis” OR “Vaskulitis” OR “FMF” OR “familiäres Mittelmeerfieber” OR “Autoinflammation” OR “AOSD.”

All 8 raters (Multimedia Appendix 1) were physicians currently completing their rheumatology fellowships. Four physicians were using Android phones and four physicians were using iPhones to individually search for the terms in the associated app stores. Raters stated no conflict of interest regarding the industry surrounding the apps being rated. Searches were performed from May 1-31, 2018.

App inclusion criteria were (1) German language, (2) availability in both app stores, (3) targeting patients or physicians as users, and (4) clearly including rheumatology or rheumatic diseases as subject matter. Exclusion criteria were (1) congress apps and (2) company apps with advertisements.

### App Evaluation

As recommended by the MARS developers, all raters viewed the training video by Stoyanov et al [9], and each app was tested for at least 10 minutes. The raters agreed on the relevance of all MARS items to this project. Before rating their assigned apps, all raters evaluated two apps selected for training purposes (previously excluded from the analysis) and discussed their results to ensure a similar understanding of the MARS items and process.

Four of the apps were rated by all raters and the remaining apps were randomly assigned to raters by creating a stratified randomization list using a virtual urn method without replacement, such that each app would be rated by two Android and two iPhone users. Apps were downloaded and rated from July 1-31, 2018. Furthermore, information was collected regarding target group, target disease, content, developer, availability of privacy policy statement, medical product status, and current app store rating. Availability of scientific studies was checked via Google, Google Scholar, PubMed, the developer website, and the app stores.

### Statistical Analysis

MARS section scores were calculated by taking the arithmetic mean of each item score in the section, while the overall score was the arithmetic mean of the section scores (excluding subjective quality). Overall scores and section scores were summarized as median and range for each app, and apps were ranked based on the median overall MARS score. We analyzed item score deviations by section and rater using a random intercept–only mixed-effects linear regression model including the individual item scores as the dependent variable, a random effects term for the rater, and nested random effects terms for the MARS section and app. Using random intercepts from this model, we estimated how the item scores in each section in each app deviated from the overall mean item score to rank and plot the importance of the sections within each app. Similarly, we plotted the random effect intercepts and respective 95% confidence intervals for raters to rank the raters by their deviation from the overall mean item score as a measure of rater bias. We analyzed the effect of rater gender and operating system on ratings by adding respective fixed effect terms to the model and reported their coefficients and 95% confidence intervals. Random intercept and fixed effect term confidence intervals spanning both sides of 0 were considered insignificant. We constructed scatter plots of MARS scores for each app and platform against their respective store ratings and calculated Pearson correlation coefficients both across platforms and separately. Finally, we analyzed interrater agreement at item, section, and overall score levels for raters from a rater sample, namely ICC2k (two-way random, average measures, absolute agreement) [12]. All data analysis was performed using the open source R software v3.5.3 (The R Foundation).

## Results

### Selection of Mobile Apps

**Figure 1:**
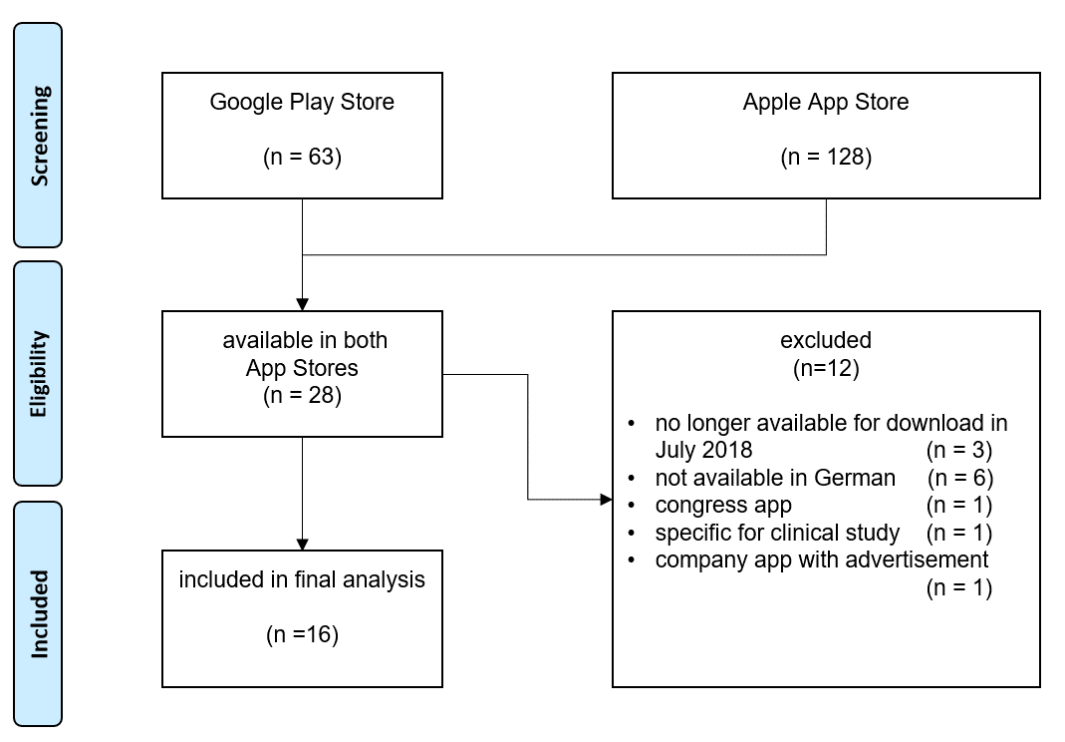
App selection process flowchart.

In total 128 and 63 apps were identified in the German App Store and Google Play, respectively. After removing duplicates and only including apps that were available in both stores, 28 apps remained. Three previously included apps were no longer available for download in July 2018 and were excluded; 9 apps were removed—6 were not available in German, one was a congress app, one a specific app for a clinical study, and one an ergo therapy advertisement app—so there remained 16 final apps for analysis (Figure 1). During the analysis, Psoriapp was no longer available in Google Play and could only be rated by iOS raters. Android rater 2 downloaded Rheumatologie Visuell but the log-in repeatedly failed. The same rater was unable to successfully download Rheuma Edu although it was available in Google Play. iOS rater 2 had the same problem with the Rheumatologie Visuell app.

### Characteristics of Mobile Apps

Tables 1 and 2 display the characteristics of the analyzed apps. Nine apps were designed for patient use, and 7 for physician use. The following rheumatologic diseases were targeted: rheumatoid arthritis (RA), psoriatic arthritis (PsA), ankylosing spondylitis (SpA), juvenile idiopathic arthritis (JIA), systemic lupus erythematosus (SLE), vasculitis, and giant cell arteritis. Thirteen apps were rheumatology specific, and 3 apps were nonspecific. All physician-targeted apps focused on education. Most of these apps were text and graphic based, focusing on guidelines. Other physician apps incorporated videos (Rheuma Edu), audio files (Meditorium), and case images (Rheumatologie Visuell). Three apps consisted of a score calculator. Eight out of 9 patient apps had a diary function of some sort. Rheuma Auszeit, the only patient app without a diary function, provided video and audio instructions for mental and physical exercises. Only 3 out of 8 diary patient apps consisted of validated disease activity questionnaires. Most apps provided a reminder function. Two out of 9 patient apps provided a service to exchange experiences via private or group messages.

Only one app, Rheuma Auszeit, was developed mainly by a patient organization; 2 apps were mainly developed by pharmaceutical companies. Five other apps were financially supported by pharmaceutical companies. All patient apps were free of charge, but 2 of the physician-targeted apps required in-app purchases to function completely. According to the associated website, the MyTherapy app has been used for an adherence study with type 2 diabetes patients; however, no details for this study were stated and the study could not be identified using Google Scholar or PubMed. Privacy policy statements were available for all apps except the ASAS App. Three patient apps were classified as medical products, all constructed by the same developer (STAR Healthcare Management GmbH).

**Table 1:** Target group, target disease, and developer of included rheumatology apps.

App	Target group	Target disease	Developer	Category
Rheuma Auszeit	Patient	RA^a^	Deutsche Rheuma-Liga Bundesverband eV	Education
Meditorium	Physician	Nonspecific	SchiLu Media UG	Education
RheumaGuide	Physician	RA^a^, PsA^b^, SpA^c^	MedMedia Verlag und Mediaservice GmbH	Education
ASAS App	Physician	SpA^c^	Assessment of SpondyloArthritis International Society	Education, calculator
RheumaLive	Patient	RA^a^	STAR Healthcare Management GmbH	Diary, reminder
Pain Companion	Patient	Nonspecific	Sanovation AG	Diary, exchange
MyTherapy	Patient	Nonspecific	Smartpatient GmbH	Diary, reminder
Psoriapp	Patient	PsA^b^	Novartis Pharma GmbH	Diary, reminder
Rheumatologie Visuell	Physician	Rheumatic	Georg Thieme Verlag KG	Education
PsALive	Patient	PsA^b^	STAR Healthcare Management GmbH	Diary, reminder
AxSpaLive	Patient	SpA^c^	STAR Healthcare Management GmbH	Diary, reminder
Lupuslog	Patient	SLE^d^	GlaxoSmithKline PLC	Diary, reminder
Rheuma Edu	Physician	Rheumatic	Pomcanys Marketing AG	Education
ANCA–Assoziierte Vaskulitiden	Physician	ANCA^e^–associated vasculitis	Börm Bruckmeier Verlag GmbH	Education
RheumaBuddy	Patient	RA^a^, JIA^f^	DAMAN P/S	Diary, exchange
Rheumatologie App	Physician	RA^a^, vasculitis	Börm Bruckmeier Verlag GmbH	Education

^a^RA: rheumatoid arthritis.

^b^PsA: psoriatic arthritis.

^c^SpA: ankylosing spondylitis.

^d^SLE: systemic lupus erythematosus.

^e^ANCA: antineutrophil cytoplasmic antibody.

^f^JIA: juvenile idiopathic arthritis.

**Table 2:** Characteristics of included rheumatology apps.

App	Technical aspects/content	Studies available	Privacy policy available	Medical product	Price
Rheuma Auszeit	Videos and audio files	No	Yes	No	Free
Meditorium	Audio files	No	Yes	No	2.99 € to 47.99 € (US $3 to $54)
RheumaGuide	Diagnostic and therapeutic guidelines, score calculator	No	Yes	No	Free
ASAS App	Diagnostic and therapeutic guidelines, score calculator	No	No	No	Free
RheumaLive	Diary^a^, medication reminder, export function	No	Yes	Yes	Free
Pain Companion	Diary, group discussion, private messages, export function	No	Yes	No	Free
MyTherapy	Tracking, medication/task reminder	No^b^	Yes	No	Free
Psoriapp	Diary, medication reminder, export function	No	Yes	No	Free
Rheumatologie Visuell	Rheumatology images	No	Yes	No	Free
AxSpaLive	Diary^a^, medication reminder, export function	No	Yes	Yes	Free
PsALive	Diary^a^, medication reminder, export function	No	Yes	Yes	Free
Lupuslog	Diary, reminder, pictures, export function, local weather	No	Yes	No	Free
Rheuma Edu	Videos	No	Yes	No	6.49 € (US $7)
ANCA–Assoziierte Vaskulitiden	Diagnostic and therapeutic guidelines	No	Yes	No	Free
RheumaBuddy	Diary, group discussion, private messages, reminder, export function	No	Yes	No	Free
Rheumatologie App	Diagnostic and therapeutic guidelines, score calculator	No	Yes	No	Free

^a^This allowed tracking of validated rheumatology-specific questionnaires.

^b^Developer website states clinical study, yet no details could be identified using Google Scholar or Pubmed.

### App Ratings

**Figure 2:**
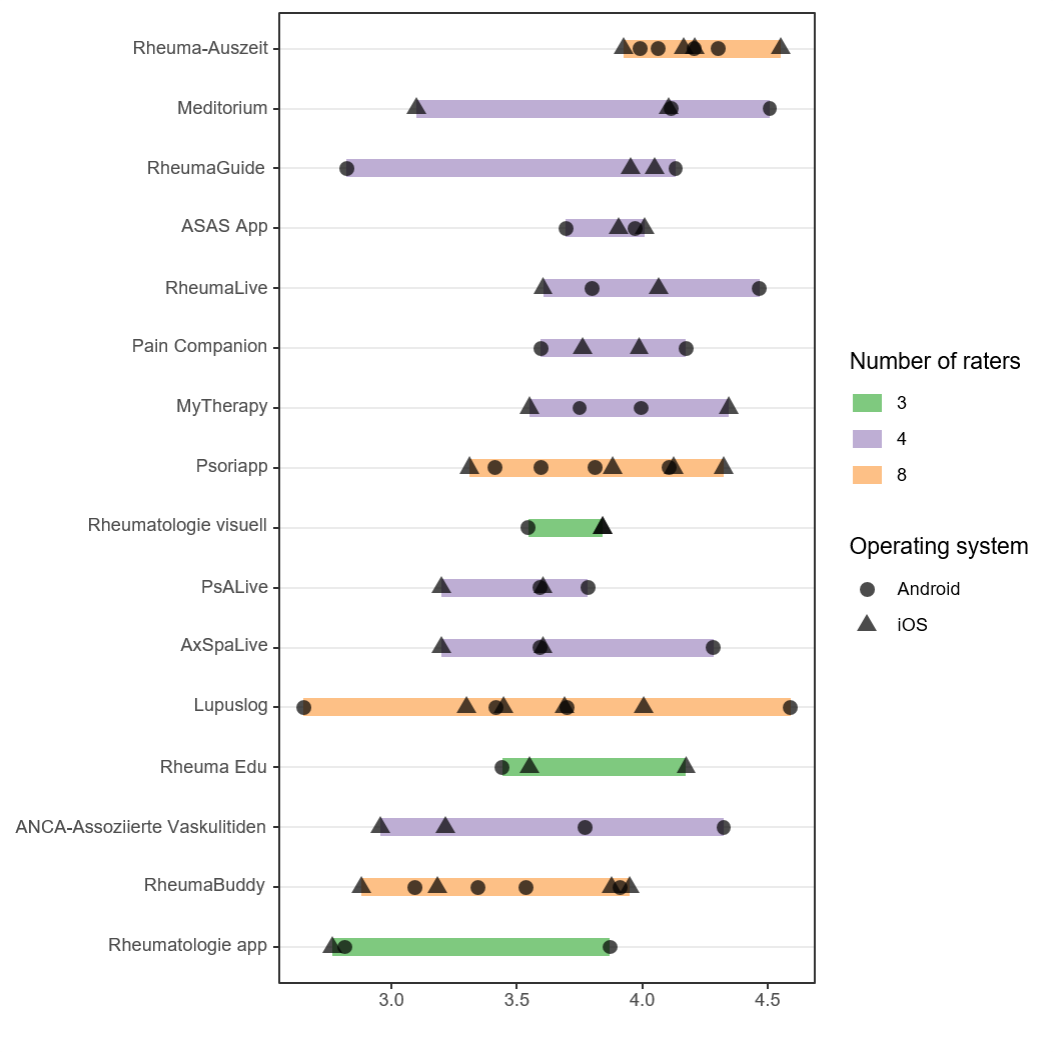
Mobile Application Rating Scale overall ratings of included apps.

The overall MARS scores ranged from 2.81 to 4.19. The apps were ranked by median overall score. The individual MARS score ratings by each rater and their range are presented in Figure 2. The individual MARS section scores by each rater and their ranges are displayed in Multimedia Appendix 2.

**Figure 3:**
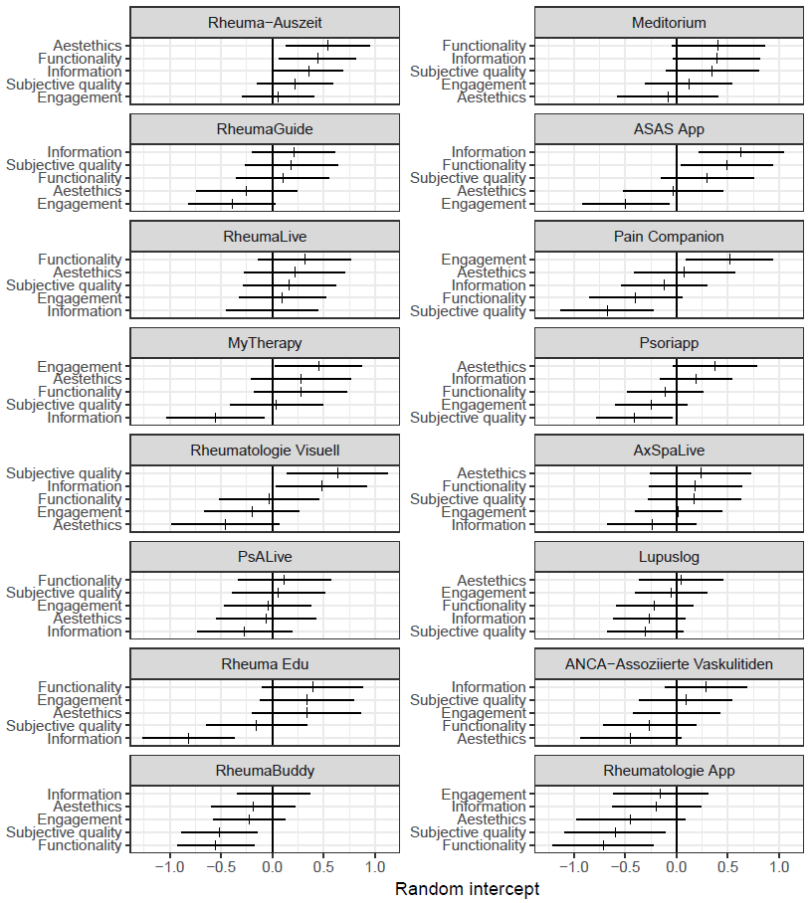
Mobile Application Rating Scale section item scores by section and app.

Random intercepts and 95% confidence intervals from the mixed-effects linear regression analysis are presented in Figure 3, summarizing the mean deviation of item scores and their 95% confidence intervals by section and their ranking within each app. This figure shows that subjective quality was the section in which item scores were most often significantly lower compared to the overall mean (Pain Companion, Psoriapp, RheumaBuddy, and Rheumatologie App). Information was the section in which item scores were most often significantly higher compared to the overall mean (Rheuma Auszeit, ASAS App, and Rheumatologie Visuell). For the aesthetics, functionality, and engagement sections there was no app with significantly lower item score deviations.

**Figure 4:**
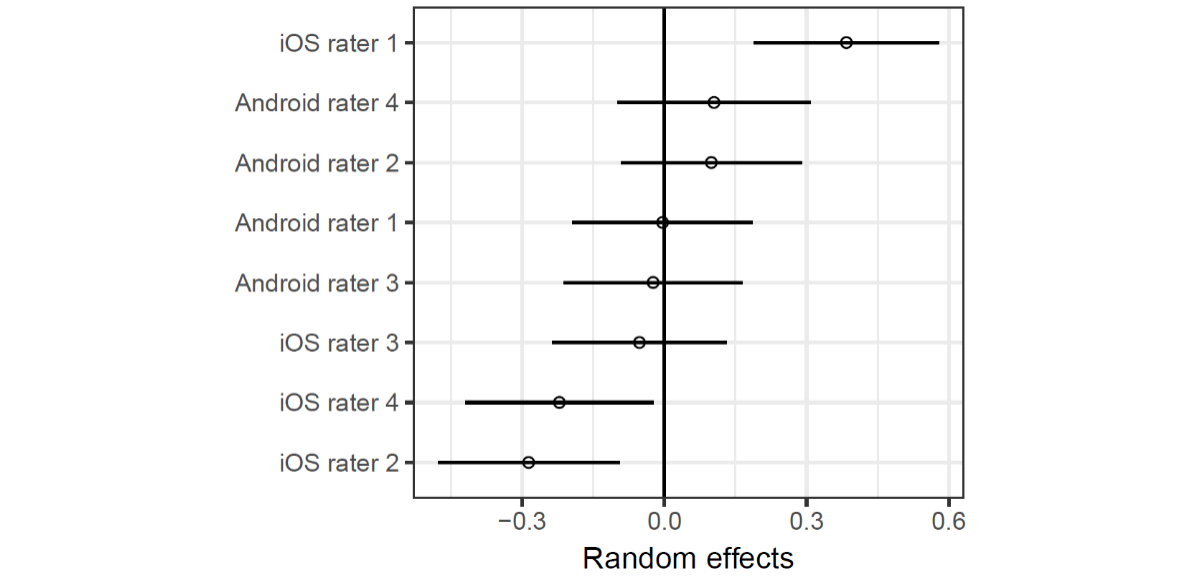
Rater deviations in item scores.

Rater agreement on overall MARS score at app level was poor and imprecise (ICC2k 0.53, 95% CI 0.08 to 0.81) whereas the interrater agreement for section scores (ICC2k 0.82, 95% CI 0.76 to 0.88) and individual item scores (ICC2k 0.84, 95% CI 0.81 to 0.86) were good. Random intercepts for observers from the mixed-effects model are presented in Figure 4.

The point estimates and confidence intervals show that 3 of the 4 iOS raters were significantly biased with respect to mean item scores either in the positive or negative direction, whereas the random intercepts for the Android raters were similar. However, adding the operating system as a fixed effect in the regression model did not seem to be associated with an overall significant difference in item scores (*β*=–0.10, 95% CI –0.44 to 0.24; *P*=.57 for iOS, compared to Android). Finally, the mixed-effects model with rater gender as a fixed effect also shows that the adjusted difference between item scores between male and female raters was small and imprecise (*β*=0.08, 95% CI –0.27 to 0.44; *P*=.64 for male gender) and does not suggest a gender effect on item scores.

MARS and app store ratings, including the range and number of ratings, are shown in Multimedia Appendix 3. App store ratings were retrieved on April 21, 2019. At the time, Psoriapp was not available in both app stores. For all apps, Google Play had more ratings than App Store. MyTherapy had by far the most ratings (24,408).

Correlation analysis between overall MARS scores of the apps and their respective store ratings was limited by the availability of store ratings. We did not find a significant correlation between MARS scores and store ratings, whether overall or grouped by operating system.

## Discussion

### Principal Findings

This analysis of German rheumatology-specific apps showed that most apps were patient focused (9 out of 16), and only a minority of rheumatic diseases were specifically targeted. For several rheumatic diseases, such as systemic sclerosis, Sjogren syndrome, Behcet disease, and familial Mediterranean fever, there were no apps available. The three apps with the highest overall MARS score included videos, audio files, and images. The inclusion of multimedia content therefore seems to be advisable.

The MARS rating itself is quite subjective, shown by the great interrater differences despite the tutorial video and test phase with discussion of the results. The poor rater agreement is likely also due to a low number of apps and a high number of raters. This result is in line with other app rating studies including more than two raters [13]. We chose not to discuss and adapt conflicting results like others [11,14] because we believe this results in a falsification of data. Studies using MARS and including only two raters often showed good interrater reliability [10]. We used MARS because it is one of the most established app rating tools; however, several tools with different weaknesses and strengths exist [7]. Interestingly, one developer created patient apps that were all certified as CE-labeled medical products for three common rheumatic diseases (RA, PsA, SpA). No other apps were found to be CE certified. However, being CE certified did not guarantee a top ranking. In order to harmonize research and increase trust and transparency, an international task force is needed to create guidelines and accepted quality criteria. These guidelines are desperately needed, as easily available quality indicators such as app store ratings only poorly reflect their true quality.

In order to increase acceptance and use among patients and health care professionals, clinical studies are also urgently needed. Only one app referred to a clinical study; however, these results could not be clearly identified using Google Scholar or PubMed. We believe that it is necessary to shift the current developer status from commercial developers toward universities and independent research institutions including patients as well as physicians. The importance of including patients is highlighted by the fact that the only app mainly developed by a patient organization had the highest MARS score (4.19/5). These findings illustrate current unmet needs hindering the use of eHealth tools despite their great potential.

Patient self-assessments via smartphone strongly correlate with rheumatologist assessments [15] and could cost effectively enhance current tight control strategies. An official, highly customizable app developed by a trusted and independent organization based on a common minimal data set would allow the creation of a holistic repository. This app would ensure maximally efficient use of resources. Due to its large user base, it would provide a powerful passive dataset for research. A role model could be the Swiss Web app, mySCQM [16], developed by Swiss Clinical Quality Management in Rheumatic Diseases. This app allows patients to enter data in between visits and share these data with their doctor and the national Swiss registry. To increase acceptance of such an app among patients, it seems advisable to also allow safe communication and file exchange between patients and their physicians. Further research to identify key components [17,18] and stakeholder preferences [5,19-21] is needed.

### Limitations

This study has some limitations. First, we only looked at app stores, and no systematic literature search was performed. For future projects, it would be time saving to use an automated process to identify and filter apps as proposed by Albrecht et al [22]; however, this process is restricted to native mobile apps. Web-based apps that are not featured in app stores are therefore not included. Due to the growing popularity of Web-based apps [23], we believe it is crucial to include these apps in future research projects.

Due to the app store approach using a limited amount of German search terms, useful rheumatology apps may have been overlooked. To facilitate app evaluation, app randomization, and data analysis, we only included apps that were available in both app stores. Another limitation is the fact that only physicians performed the app rating, although most of the apps were created for patient use. As already suggested by others [13,24], future research should include patients.

Due to lack of expertise and resources, we only checked the data security very briefly in terms of presence of a data policy statement, password protection, and log-in requirement. A professional in-depth security check should be applied to identify any risks, as mobile apps often do not follow data protection laws [25] and could be potentially harmful for the end user [26]. Finally, it should be mentioned that due to the rapid speed of mobile app development, this review might already be out of date once published. A main limitation of the MARS score itself is that to our knowledge there is no clear definition of a high-quality app, and its meaning often varies [11,27].

### Comparison With Prior Work

A major strength of this study lies in its ability to guide recommendations of apps by rheumatologists for their patients. To our knowledge, no review and analysis of mobile apps in rheumatology available in German app stores has been carried out yet. In contrast to other studies [13,28] using MARS, we identified apps targeting patients as well as physicians. Furthermore, apps were tested on iOS and Android platforms to identify usability differences. To our knowledge, no previous study using MARS had as many raters as this study did. We tried to include many raters to better represent different subjective perspectives and pick up any interrater rating weaknesses of the MARS.

The low number of recommendable rheumatology-specific apps found in the previous survey [5] can now largely be explained by the lack of German rheumatology-specific apps in general (16 identified apps in total) and their heterogeneous quality. However, in contrast to a previous rheumatology app review [10], we identified one patient and one physician app with promising overall MARS scores (>4/5).

This study exposes the lack of reliable studies for mobile apps in general [29] and specifically in rheumatology [10]. Similarly, in this work we observed a wide range of MARS scores reflecting heterogeneous quality. Grainger et al [10] reported that 6 out of 11 patient-targeted apps allowed data sharing. In our analysis, this was the case for 8 out of 9 patient-targeted apps.

The lack of academic app developers reported by Salazar et al [11] is supported by our work. In accordance with prior publications [11,27], there was no strong correlation of app store ratings and MARS ratings. App store ratings therefore seem to be a poor quality indicator. In our analysis, 89% (8/9) of the patient apps had a symptom tracking function. In a previous publication [17] focusing on rheumatoid arthritis, this was only the case for 50% of the apps.

Noticeably, Rheuma Auszeit, the only app mainly developed by a patient organization, had the best MARS score and lowest rater standard deviation. This highlights the importance and success of including patients in the app development process, as stressed by Grainger et al [10]. Interestingly, this was the only patient app not containing a diary function. The name of the app is translated as rheuma timeout, implying that the goal of this app is exactly the opposite of tracking pain. This could be a main cause of adherence problems with patient apps, as patients are constantly reminded of their disease and limitations.

Based on our study findings, we established 10 recommendations (Multimedia Appendix 4) that might direct developers to create better apps that maximize patient and physician satisfaction.

### Conclusion

To our knowledge, this is the first study to systematically identify and evaluate mobile apps in rheumatology for patients and physicians available in German app stores. App quality, origin, and amount of evidence was heterogeneous. Brief descriptions and recommendations were compiled to provide ready-to-use, useful information for potential users and developers.

We recommend continual evaluation of mobile apps based on automatic crawling techniques; quality evaluations by users (patients and physicians); and supporting cost-effectiveness studies to enhance awareness, use, and potential benefit. Furthermore, we would like to emphasize the importance of research institutes and academics as data recipients and partners in app development. Only then can truly powerful data analysis and insights be collected and used for scientific research.

To maximize the great eHealth potential in rheumatology, a close collaboration of patients, rheumatologists, developers, and industry is needed. To avoid redundant work and save time, international and national eHealth consortiums and collaborations are needed to create guidelines and recommendations.

## Appendix

Multimedia Appendix 1 Rater gender, hardware and software.

Multimedia Appendix 2 Individual Mobile Application Rating Scale section ratings of included apps.

Multimedia Appendix 3 Mobile Application Rating Scale and app store ratings of included apps.

Multimedia Appendix 4 Recommendations for developers of mobile rheumatology apps.

## Abbreviations

ANCA: antineutrophil cytoplasmic antibodies
ICC2k: two-way random, average measures, absolute agreement
JIA: juvenile idiopathic arthritis
MARS: Mobile Application Rating Scale
PsA: psoriatic arthritis
RA: rheumatoid arthritis
SLE: systemic lupus erythematosus
SpA: ankylosing spondylitis

## References

[1] Kataria S, Ravindran V (2018). Digital health: a new dimension in rheumatology patient care. Rheumatol Int.

[2] Burmester G (2018). Rheumatology 4.0: big data, wearables and diagnosis by computer. Ann Rheum Dis.

[3] Ronicke S, Hirsch MC, Türk E, Larionov K, Tientcheu D, Wagner AD (2019). Can a decision support system accelerate rare disease diagnosis? Evaluating the potential impact of Ada DX in a retrospective study. Orphanet J Rare Dis.

[4] Gossec L, Guyard F, Leroy D, Lafargue T, Seiler M, Jacquemin C, Molto A, Sellam J, Foltz V, Gandjbakhch F, Hudry C, Mitrovic S, Fautrel B, Servy H (2018). Detection of flares by decrease in physical activity, collected using wearable activity trackers, in rheumatoid arthritis or axial spondyloarthritis: an application of machine-learning analyses in rheumatology. Arthritis Care Res (Hoboken).

[5] Knitza J, Vossen D, Geffken I, Krusche M, Meyer M, Sewerin P, Kleyer A, Hueber AJ (2018). [Use of medical apps and online platforms among German rheumatologists: results of the 2016 and 2018 DGRh conference surveys and research conducted by rheumadocs]. Z Rheumatol.

[6] Albrecht U, Hillebrand U, von Jan U (2018). Relevance of trust marks and CE labels in German-language store descriptions of health apps: analysis. JMIR Mhealth Uhealth.

[7] Nouri R, R Niakan Kalhori S, Ghazisaeedi M, Marchand G, Yasini M (2018). Criteria for assessing the quality of mHealth apps: a systematic review. J Am Med Inform Assoc.

[8] Wyatt JC (2018). How can clinicians, specialty societies and others evaluate and improve the quality of apps for patient use?. BMC Med.

[9] Stoyanov SR, Hides L, Kavanagh DJ, Zelenko O, Tjondronegoro D, Mani M (2015). Mobile app rating scale: a new tool for assessing the quality of health mobile apps. JMIR Mhealth Uhealth.

[10] Grainger R, Townsley H, White B, Langlotz T, Taylor W (2017). Apps for people with rheumatoid arthritis to monitor their disease activity: a review of apps for best practice and quality. JMIR Mhealth Uhealth.

[11] Salazar A, de Sola H, Failde I, Moral-Munoz JA (2018). Measuring the quality of mobile apps for the management of pain: systematic search and evaluation using the mobile app rating scale. JMIR Mhealth Uhealth.

[12] Shrout PE, Fleiss JL (1979). Intraclass correlations: uses in assessing rater reliability. Psychol Bull.

[13] Powell AC, Torous J, Chan S, Raynor GS, Shwarts E, Shanahan M, Landman AB (2016). Interrater reliability of mHealth app rating measures: analysis of top depression and smoking cessation apps. JMIR Mhealth Uhealth.

[14] Bardus M, van Beurden SB, Smith JR, Abraham C (2016). A review and content analysis of engagement, functionality, aesthetics, information quality, and change techniques in the most popular commercial apps for weight management. Int J Behav Nutr Phys Act.

[15] Walker UA, Mueller RB, Jaeger VK, Theiler R, Forster A, Dufner P, Ganz F, Kyburz D (2017). Disease activity dynamics in rheumatoid arthritis: patients' self-assessment of disease activity via WebApp. Rheumatology (Oxford).

[16] Swiss Clinical Quality Management in Rheumatic Diseases.

[17] Luo D, Wang P, Lu F, Elias J, Sparks JA, Lee YC (2019). Mobile apps for individuals with rheumatoid arthritis: a systematic review. J Clin Rheumatol.

[18] Waite-Jones JM, Majeed-Ariss R, Smith J, Stones SR, Van Rooyen V, Swallow V (2018). Young people’s, parents’, and professionals’ views on required components of mobile apps to support self-management of juvenile arthritis: qualitative study. JMIR Mhealth Uhealth.

[19] van der Vaart R, Drossaert CHC, Taal E, van de Laar MA (2011). Patient preferences for a hospital-based rheumatology Interactive Health Communication Application and factors associated with these preferences. Rheumatology (Oxford).

[20] Mollard E, Michaud K (2018). A mobile app with optical imaging for the self-management of hand rheumatoid arthritis: pilot study. JMIR Mhealth Uhealth.

[21] Revenäs A, Opava CH, Martin C, Demmelmaier I, Keller C, Åsenlöf P (2015). Development of a web-based and mobile app to support physical activity in individuals with rheumatoid arthritis: results from the second step of a co-design process. JMIR Res Protoc.

[22] Albrecht U, Hasenfuß G, von Jan U (2018). Description of cardiological apps from the German app store: semiautomated retrospective app store analysis. JMIR Mhealth Uhealth.

[23] Turner-McGrievy G, Hales S, Schoffman D, Valafar H, Brazendale K, Weaver R, Beets M, Wirth M, Shivappa N, Mandes T, Hébert J, Wilcox S, Hester A, McGrievy M (2017). Choosing between responsive-design websites versus mobile apps for your mobile behavioral intervention: presenting four case studies. Transl Behav Med.

[24] Baptista S, Oldenburg B, O'Neil A (2017). Response to “Development and validation of the user version of the Mobile Application Rating Scale (uMARS)”. JMIR Mhealth Uhealth.

[25] Papageorgiou A, Strigkos M, Politou E, Alepis E, Solanas A, Patsakis C (2018). Security and privacy analysis of mobile health applications: the alarming state of practice. IEEE Access.

[26] Lewis TL, Wyatt JC (2014). mHealth and mobile medical Apps: a framework to assess risk and promote safer use. J Med Internet Res.

[27] Terhorst Y, Rathner E, Baumeister H, Sander L (2018). [“Hilfe aus dem App-Store?”: Eine systematische Übersichtsarbeit und Evaluation von Apps zur Anwendung bei Depressionen]. Verhaltenstherapie.

[28] Santo K, Richtering s, Chalmers j, Thiagalingam a, Chow c, Redfern j (2016). Mobile phone apps to improve medication adherence: a systematic stepwise process to identify high-quality apps. JMIR Mhealth Uhealth.

[29] Byambasuren O, Sanders S, Beller E, Glasziou P (2018). Prescribable mHealth apps identified from an overview of systematic reviews. npj Digital Med.

