# Supplementary material for: German Mobile Apps in Rheumatology: Review and Analysis Using the Mobile Application Rating Scale (MARS)
Source: JMIR Mhealth Uhealth. 2019 Aug 5;7(8):e14991. doi: 10.2196/14991 (PMC6699116; doi:10.2196/14991)
Supplement: Multimedia Appendix 1 [file mhealth_v7i8e14991_app1.pdf]

Rater	Gender	Hardware	Software
iOS rater 1	male	Apple iPhone 7	iOS 12.0.0
iOS rater 2	male	Apple iPhone 6	iOS 12.0.1
iOS rater 3	male	Apple iPhone SE	iOS 12.0.1
iOS rater 4	female	Apple iPhone X	iOS 12.0.0
Android rater 1	male	LG G4 Stylus	Android 6.0
Android rater 2	male	Samung Galaxy S7	Android 6.0
Android rater 3	female	Huawei Mate 20 Pro	Android 6.0
Android rater 4	female	Sony Experia Z3	Android 6.0
