# Supplementary figures and images for: German Mobile Apps in Rheumatology: Review and Analysis Using the Mobile Application Rating Scale (MARS)
Source: JMIR Mhealth Uhealth. 2019 Aug 5;7(8):e14991. doi: 10.2196/14991 (PMC6699116; doi:10.2196/14991)

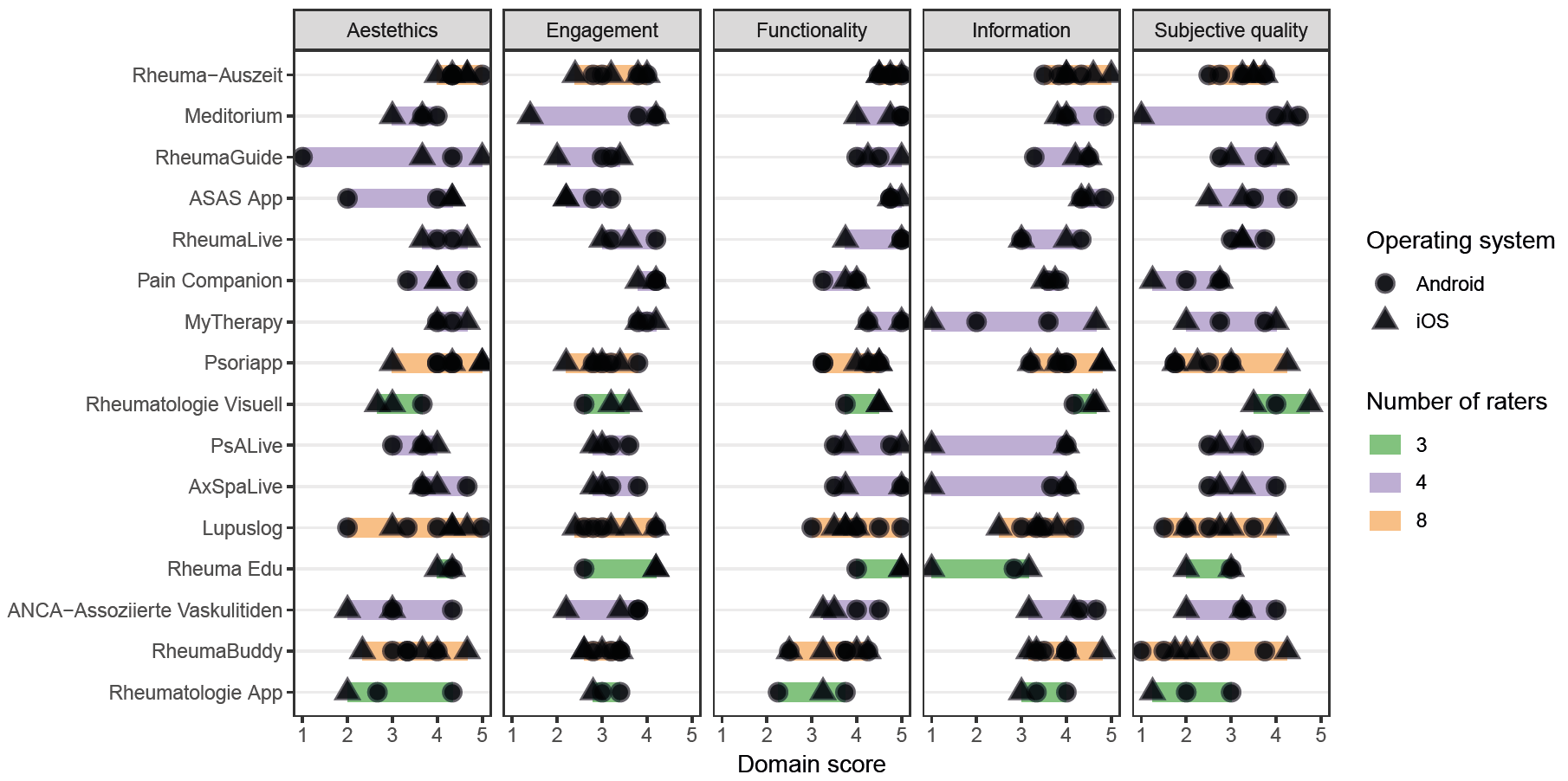

Supplement: Multimedia Appendix 2 [file mhealth_v7i8e14991_app2.png]

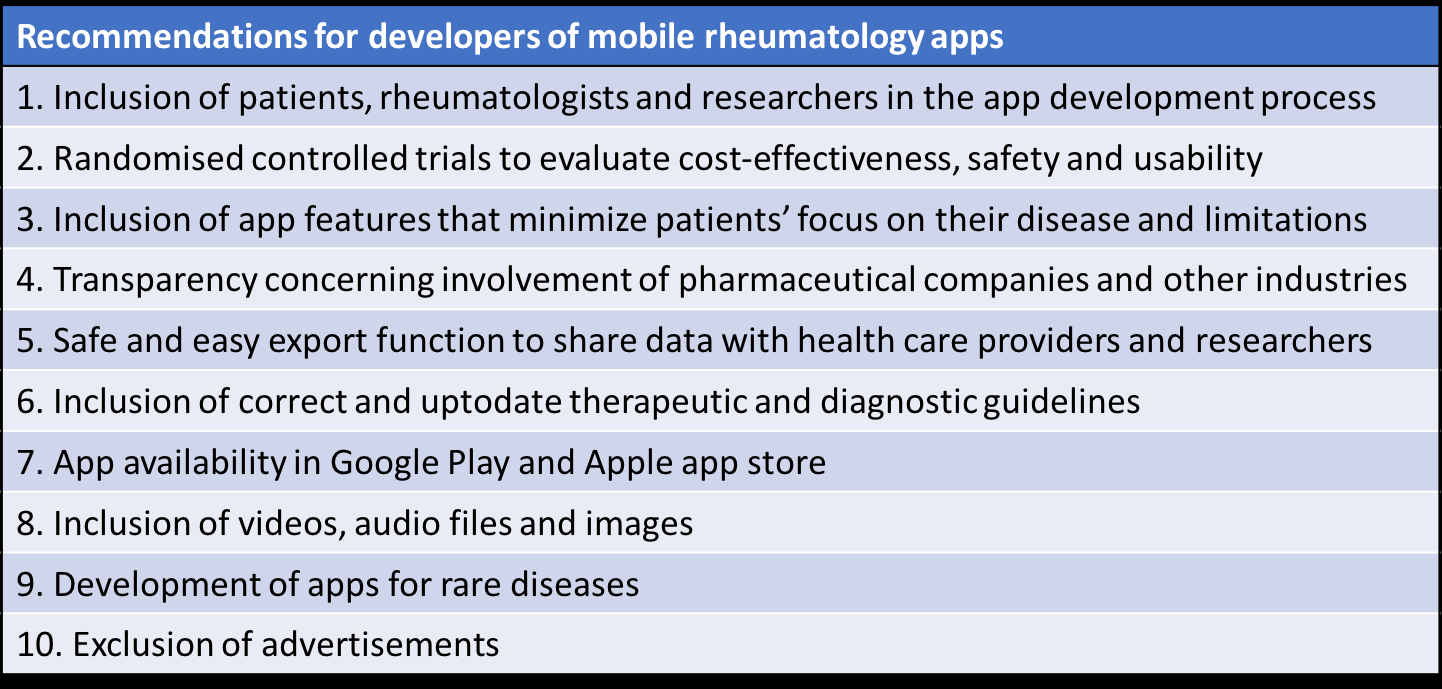

Supplement: Multimedia Appendix 4 [file mhealth_v7i8e14991_app4.png]
