# Supplementary material for: German Mobile Apps in Rheumatology: Review and Analysis Using the Mobile Application Rating Scale (MARS)
Source: JMIR Mhealth Uhealth. 2019 Aug 5;7(8):e14991. doi: 10.2196/14991 (PMC6699116; doi:10.2196/14991)
Supplement: Multimedia Appendix 3 [file mhealth_v7i8e14991_app3.pdf]

App	MARS[a] score, median (range)	iOS raters, N	Android raters, N	MARS[a] section scores, median (range)					Google Play Store ratings[b], mean (N)	iTunes Store ratings[b], mean (N)
				Aestethics	Engagement	Functionality	Information	Subjective quality		
**Rheuma-Auszeit**	4.19 (3.92 - 4.55)	4	4	4.33 (4.00 - 5.00)	3.50 (2.40 - 4.00)	4.75 (4.50 - 5.00)	4.00 (3.50 - 5.00)	3.38 (2.50 - 3.75)	4.4 (40)	4.4 (7)
**Meditorium**	4.11 (3.10 - 4.51)	2	2	3.67 (3.00 - 4.00)	4.00 (1.40 - 4.20)	4.88 (4.00 - 5.00)	4.00 (3.80 - 4.83)	4.12 (1.00 - 4.50)	4.6 (263)	4.7 (97)
**RheumaGuide**	4.00 (2.82 - 4.13)	2	2	4.00 (1.00 - 5.00)	3.10 (2.00 - 3.40)	4.38 (4.00 - 5.00)	4.35 (3.29 - 4.50)	3.38 (2.75 - 4.00)	4.0 (4)	na[c]
**ASAS App**	3.94 (3.70 - 4.01)	2	2	4.17 (2.00 - 4.33)	2.50 (2.20 - 3.20)	4.75 (4.75 - 5.00)	4.42 (4.33 - 4.83)	3.38 (2.50 - 4.25)	4.4 (25)	5.0 (1)
**RheumaLive**	3.93 (3.60 - 4.47)	2	2	4.17 (3.67 - 4.67)	3.40 (3.00 - 4.20)	5.00 (3.75 - 5.00)	3.50 (3.00 - 4.33)	3.25 (3.00 - 3.75)	3.8 (5)	5.0 (2)
**Pain Companion**	3.88 (3.60 - 4.17)	2	2	4.00 (3.33 - 4.67)	4.20 (3.80 - 4.20)	3.88 (3.25 - 4.00)	3.67 (3.50 - 3.83)	2.38 (1.25 - 2.75)	4.1 (378)	3.4 (20)
**MyTherapy**	3.87 (3.55 - 4.35)	2	2	4.17 (4.00 - 4.67)	3.90 (3.80 - 4.20)	4.62 (4.25 - 5.00)	2.80 (1.00 - 4.67)	3.25 (2.00 - 4.00)	4.7 (24.408)	4.8 (1.450)
**Psoriapp**	3.85 (3.31 - 4.33)	4	4	4.33 (3.00 - 5.00)	3.00 (2.20 - 3.80)	4.25 (3.25 - 4.50)	3.90 (3.20 - 4.80)	2.38 (1.75 - 4.25)	na[d]	na[d]
**Rheumatologie visuell**	3.84 (3.55 - 3.84)	2	1	3.00 (2.67 - 3.67)	3.20 (2.60 - 3.60)	4.50 (3.75 - 4.50)	4.60 (4.17 - 4.67)	4.00 (3.50 - 4.75)	3.0 (2)	na[c]
**AxSpaLive**	3.60 (3.20 - 4.28)	2	2	3.83 (3.67 - 4.67)	3.10 (2.80 - 3.80)	4.38 (3.50 - 5.00)	3.83 (1.00 - 4.00)	3.00 (2.50 - 4.00)	3.0 (3)	5.0 (1)
**PsALive**	3.60 (3.20 - 3.78)	2	2	3.67 (3.00 - 4.00)	3.10 (2.80 - 3.60)	4.25 (3.50 - 5.00)	4.00 (1.00 - 4.00)	3.00 (2.50 - 3.50)	1.5 (2)	5.0 (1)
**Lupuslog**	3.57 (2.65 - 4.59)	4	4	4.17 (2.00 - 5.00)	3.10 (2.40 - 4.20)	3.88 (3.00 - 5.00)	3.37 (2.50 - 4.17)	2.62 (1.50 - 4.00)	4.5 (11)	4.0 (1)
**Rheuma Edu**	3.55 (3.44 - 4.17)	2	1	4.33 (4.00 - 4.33)	4.20 (2.60 - 4.20)	5.00 (4.00 - 5.00)	2.83 (1.00 - 3.17)	3.00 (2.00 - 3.00)	2.8 (5)	na[c]
**ANCA-Assoziierte Vaskulitiden**	3.49 (2.95 - 4.33)	2	2	3.00 (2.00 - 4.33)	3.60 (2.20 - 3.80)	3.75 (3.25 - 4.50)	4.23 (3.17 - 4.67)	3.25 (2.00 - 4.00)	4.0 (6)	na[c]
**RheumaBuddy**	3.44 (2.88 - 3.95)	4	4	3.50 (2.33 - 4.67)	3.10 (2.60 - 3.40)	3.75 (2.50 - 4.25)	3.75 (3.17 - 4.80)	2.12 (1.00 - 4.25)	4.0 (34)	5.0 (1)
**Rheumatologie app**	2.81 (2.76 - 3.87)	1	2	2.67 (2.00 - 4.33)	3.00 (2.80 - 3.40)	3.25 (2.25 - 3.75)	3.33 (3.00 - 4.00)	2.00 (1.25 - 3.00)	3.0 (4)	2.3 (3)

[a]Mobile App Rating Scale

[b]App Store ratings retrieved on April 21st 2019

[c]No App Store ratings available

[d]App not found in App Store as of April 21st 2019
